# Microbes, metabolites and muscle: Is the gut–muscle axis a plausible therapeutic target in Duchenne muscular dystrophy?

**DOI:** 10.1113/EP091063

**Published:** 2023-06-03

**Authors:** Anthony L. Marullo, Ken D. O'Halloran

**Affiliations:** ^1^ Department of Physiology, School of Medicine, College of Medicine and Health University College Cork Cork Ireland

**Keywords:** Duchenne muscular dystrophy, gut microbial therapies, gut–muscle axis, metabolic signalling

## Abstract

Skeletal muscle is the largest metabolic organ making up ∼50% of body mass. Because skeletal muscle has both metabolic and endocrine properties, it can manipulate the microbial populations within the gut. In return, microbes exert considerable influence on skeletal muscle via numerous signalling pathways. Gut bacteria produce metabolites (i.e., short chain fatty acids, secondary bile acids and neurotransmitter substrates) that act as fuel sources and modulators of inflammation, influencing host muscle development, growth and maintenance. The reciprocal interactions between microbes, metabolites and muscle establish a bidirectional gut–muscle axis. The muscular dystrophies constitute a broad range of disorders with varying disabilities. In the profoundly debilitating monogenic disorder Duchenne muscular dystrophy (DMD), skeletal muscle undergoes a reduction in muscle regenerative capacity leading to progressive muscle wasting, resulting in fibrotic remodelling and adipose infiltration. The loss of respiratory muscle in DMD culminates in respiratory insufficiency and eventually premature death. The pathways contributing to aberrant muscle remodelling are potentially modulated by gut microbial metabolites, thus making them plausible targets for pre‐ and probiotic supplementation. Prednisone, the gold standard therapy for DMD, drives gut dysbiosis, inducing a pro‐inflammatory phenotype and leaky gut barrier contributing to several of the well‐known side effects associated with chronic glucocorticoid treatment. Several studies have observed that gut microbial supplementation or transplantation exerts positive effects on muscle, including mitigating the side effects of prednisone. There is growing evidence in support of the potential for an adjunctive microbiota‐directed regimen designed to optimise gut–muscle axis signalling, which could alleviate muscle wasting in DMD.

## Abstract (highlights)

- **What is the topic of this review?**
  The contribution of gut microbial signalling to skeletal muscle maintenance and development and identification of potential therapeutic targets in progressive muscle degenerative diseases such as Duchenne muscular dystrophy.
- **What advances does it highlight?**
  Gut microbe‐derived metabolites are multifaceted signalling molecules key to muscle function, modifying pathways contributing to skeletal muscle wasting, making them a plausible target for adjunctive therapy in muscular dystrophy.

## Graphical abstract

Interactions within the large intestine between the gut microbiota and host epithelium, as well as between the microbiome and substrates (digested and non‐digestible molecules) produce metabolites that affect downstream pathways influencing muscle development and maintenance. Similarly, exercise/sedentary behaviour can feed back and modulate microbial populations within the colon forming a bidirectional gut–muscle axis. Recent evidence suggests that manipulation of both the substrates and microbial populations in the colon can mitigate muscular dystrophy‐related atrophy, establishing novel adjunctive therapeutic strategies.

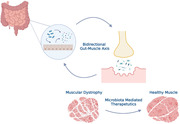

## 1 GUT–MUSCLE AXIS

Skeletal muscle is the largest metabolic organ making up ∼50% of total body mass (Valentino et al., 2021). While primarily associated with locomotion, skeletal muscle is also responsible for other vital functions, including influencing bone density, insulin‐stimulated glucose uptake, fatty acid oxidation and whole‐body protein metabolism (Lahiri et al., 2019; Valentino et al., 2021). Additionally, skeletal muscle acts as an endocrine organ eliciting systemic effects via the release of growth factors and cytokines (Pedersen et al., 1999). Both the metabolic and endocrine nature of skeletal muscle allow communication with other systems such as the digestive system, including the microbial population that resides within the gut.

Skeletal muscle mass and composition is highly plastic, regulated by a balance of muscle protein synthesis and breakdown that is affected by consumption of food (i.e., nutrient supply), physical activity/inactivity, illness and inflammation (Lochlainn et al., 2018). If the rate of protein synthesis is reduced, or breakdown/degradation is increased, muscle wasting occurs (Bindels & Delzenne, 2013). The relationships between exercise and muscle protein recruitment/synthesis, as well as sedentary behaviour/disuse and loss, are well studied. Increased physical activity enhances muscle protein synthesis, while a sedentary lifestyle can contribute to muscle wasting (atrophy).

### 1.1 Human studies

Studies investigating the gut microbial populations of athletes report that exercise positively correlates with a larger diversity of beneficial commensal gut microbial populations. Not only is a higher diversity beneficial, but stability of the microbial population has also proven to be advantageous (Furber et al., 2022). For example, in comparisons between a group of professional rugby players and a control group of healthy similar non‐sport playing individuals (matched for age and body mass index), the athletes had a higher diversity of beneficial microbes (Clarke et al., 2014). Similarly, in a study comparing amateur and professional cyclists, the professional cyclists had larger populations of microbes associated with energy and carbohydrate metabolism, facilitating the energy requirements demanded by more intense exercise (Petersen et al., 2017). However, in these types of studies it is difficult to parse out the independent contributions of exercise and diet.

**FIGURE 1:**
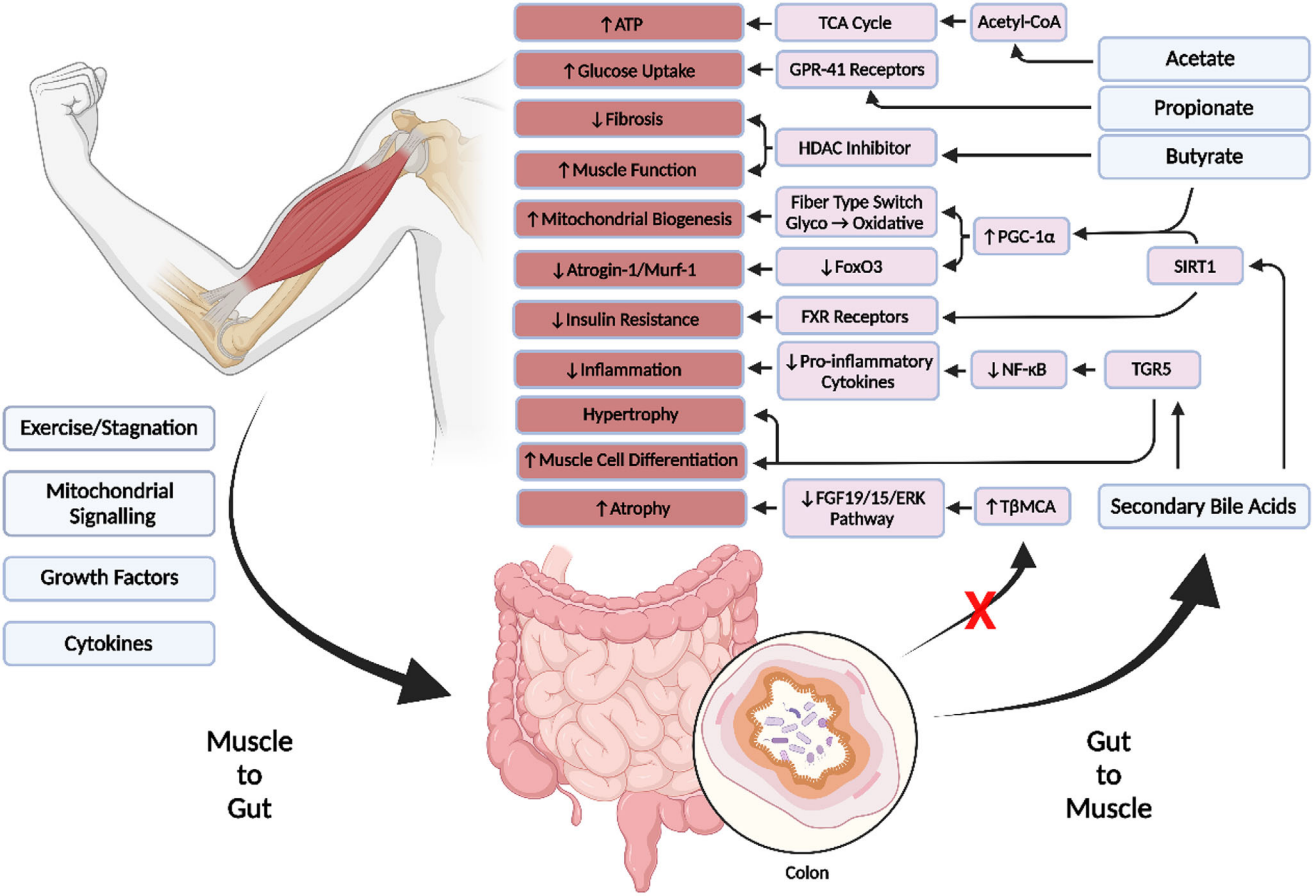
Summary of the bidirectional gut–muscle axis. Primary factors from both muscle and microbiota are in blue boxes, signalling pathways in pink, and effects in muscle in terracotta. Exercise and muscle use can alter gut microbial status via endocrine signalling. The gut can in turn produce metabolites that influence skeletal muscle development in a positive manner, while an absence of microbial influence on signalling molecules can result in a lack of development and atrophy.

While studies illustrating a direct correlation between exercise and gut microbial populations are abundant (Barton et al., 2018; Clarke et al., 2014; Mach & Fuster‐Botella, 2017; Petersen et al., 2017), the exact mechanisms by which exercise shapes microbial status remain elusive. One potential mechanism could be through mitochondrial crosstalk whereby muscle mitochondria induce innate immune responses or influence intestinal functional effector cells (e.g., immune cells, epithelial cells and enterochromaffin cells) via production of reactive oxygen species (ROS) and reactive nitrogen species (RNS), thereby altering signalling within the digestive tract (Clark & Mach, 2017). A summary of the way muscles can affect gut microbial populations is given in Figure 1. Contracting skeletal muscle also has the capacity to produce myokines, cytokines and proteins that elicit autocrine, paracrine or endocrine effects (Pedersen et al., 2003). The myokine that has been studied the most is interleukin (IL)‐6. Elevated systemic IL‐6 can affect the gut environment by stimulating intestinal L‐cells, causing secretion of glucagon‐like peptide 1 (GLP‐1) (Ellingsgaard et al., 2011). GLP‐1 is an incretin hormone that acts on β‐cells of the pancreas to enhance insulin secretion, and functions to decrease intestinal motility, and enhance satiety, thus promoting nutrient availability (Ellingsgaard et al., 2011). Alterations to gut motility have long been understood to affect gut microbial populations (Vantrappen et al., 1977). The capacity for myokines to affect the gut environment may extend beyond IL‐6; however current research is limited and must be expanded upon in the future.

Additionally, the relationship between gut microbial profile and individual status extends beyond exercise. Individuals who experience age‐related sarcopenia also have unique gut microbial profiles noting a decrease in short chain fatty acid (SCFA)‐producing bacteria (Claesson et al., 2012; Kang et al., 2021). While age‐related changes in the gut microbiome are primarily a result of lifestyle changes (i.e., diet, exercise and medications), similar observations have been made in clinical studies looking at the gut microbial status in different cachexia cohorts (Hakozaki et al., 2022; Ni et al., 2021; Ubachs et al., 2021).

Human studies allow for a glimpse at potential relationships between individual status and gut microbial populations; however, they are not without caveats. It is difficult to control for diet and other lifestyle factors. Therefore, it is advantageous to use animal models to obtain a more intimate and controlled look at the interplay between gut microbial signalling and muscle. The following section will explore animal studies offering a more in‐depth look at microbial signalling and associated pathways in the context of skeletal muscle.

## 2 METABOLIC SIGNALLING

The first study to suggest the presence of a gut–muscle axis was a pilot study investigating the mechanisms underlying the resistance to diet‐induced obesity in germ‐free mice (Bäckhed et al., 2007). The study discovered that germ‐free mice were protected from weight gain during consumption of a high‐fat and high‐sugar Western diet, presenting lean phenotypes with increased skeletal muscle. This phenotype was due to elevated fasting‐induced adipocyte factor (Fiaf) expression in the intestines (Bäckhed et al., 2007). Previous studies have observed that gut microbial interactions suppress expression of Fiaf (Bäckhed et al., 2004) Fiaf is a glycoprotein that regulates lipid metabolism and adiposity via fatty acid oxidation through AMP‐activated protein kinase (AMPK) activation in skeletal muscle; it attenuates muscle lipid uptake by inhibiting lipoprotein lipase activity (Nay et al., 2019). AMPK is a heterotrimeric enzyme that functions as a ‘fuel gauge’, activating glucose and fatty acid uptake and oxidation when cellular energy is low (Bindels & Delzenne, 2013). Fiaf is also associated with increased peroxisome proliferator‐activated receptor γ coactivator 1α (PGC‐1α) expression. PGC‐1α regulates mitochondrial biogenesis and oxidative metabolism by promoting fibre‐type switching from glycolytic to oxidative fibres, playing a vital role in skeletal muscle health (Bindels & Delzenne, 2013). PGC‐1α also suppresses FOXO3, a transcription factor that upregulates expression of atrophy‐related ubiquitin ligases atrogin‐1 and MuRF‐1 (atrogenes), thereby protecting against muscular atrophy (Sandri et al., 2006). The results of the study suggest that the gut microbiome can alter body composition via regulation of host bioenergetic pathways in mouse models.

Mouse studies that intentionally disrupt the gut microbial population have further corroborated that the gut microbiome can alter body composition. In one study, antibiotic‐induced dysbiosis in mouse models impaired skeletal muscle adaptation to exercise, blunted hypertrophy and induced a muscle fibre shift (Valentino et al., 2021). In a separate study, antibiotic treatment in mice resulted in skeletal muscle atrophy and changes in the expression of genes involved in the muscle peripheral circadian rhythm and metabolic regulation (Manickam et al., 2018). The disruptive effect of the gut microbiome on body composition and skeletal muscle is evident, which has motivated further studies investigating metabolic signalling within the gut–muscle axis.

### 2.1 Short chain fatty acid signalling

SCFAs (e.g., acetate, butyrate and propionate) have been a primary focus in the delineation of gut microbial signalling. Thus, predictably, they also play a central role in the gut–muscle axis. SCFAs are produced by microbes in the large intestine via fermentation of non‐digestible carbohydrates that remain after digestion of different food sources (e.g., cereals, fruits and vegetables; Francisco & Malagelada, 2003; Rastelli et al., 2019). Skeletal muscle has considerable redundancy in the maintenance of ATP requirements. Contracting skeletal muscle can utilise numerous substrates to generate ATP (e.g., creatine phosphate, muscle glycogen, plasma glucose and free fatty acids; Okamoto et al., 2019). The SCFA acetate can be converted to acetyl‐CoA by acetyl‐CoA synthetase 2 serving as a substrate in fatty acid synthesis and/or in the tricarboxylic acid (TCA) cycle, thereby contributing to multiple ATP‐producing pathways (Okamoto et al., 2019). One study observed that antibiotic treatment of mice lowered serum acetate levels resulting in impaired endurance exercise performance, suggesting that acetate is an important energy source for skeletal muscle (Okamoto et al., 2019). However, high acetate concentrations have also been associated with increased insulin resistance and obesity, which are both positively correlated with muscle anabolic resistance (Goffredo et al., 2016; Perry et al., 2016). These findings reinforce that molecular signalling is complex, affecting numerous pathways.

The SCFA butyrate has also been determined to play numerous roles within the host system. Butyrate is a primary fuel source for colonocytes within the enteric nervous system. Butyrate is transported into colonocyte mitochondria where it undergoes β‐oxidation to acetyl‐CoA, which enters the TCA cycle resulting in the reduction of NAD^+^ to NADH, which enters the electron transport chain producing ATP and CO_2_ (Donohoe et al., 2011). One study observed that antibiotic administration lowered luminal butyrate levels, resulting in colonocytes shifting to anaerobic glycolysis (Zarrinpar et al., 2018). The metabolic shift altered host glucose homeostasis by lowering serum glucose levels, increasing insulin sensitivity and hepatic gluconeogenesis (Zarrinpar et al., 2018). A separate study involving mouse models observed a reduction in skeletal muscle glycogen storage after antibiotic treatment, further contributing to a reduction in muscle endurance. The authors postulated that the shift in metabolism (i.e., butyrate consumption to glucose) contributed to a decrease in glycogen stores as glycogen storage requires both carbohydrate availability and uptake (Nay et al., 2019). Butyrate is also a general histone deacetylase (HDAC) inhibitor. HDACs are enzymes that remove the acetyl group from lysine residues, making DNA less likely to be transcribed (Walsh et al., 2015). HDACs play crucial roles in skeletal muscle development and maintenance. Class II HDACs suppress myoblast differentiation regulating myoblast activity through interactions with the transcription factor myocyte enhancer factor 2 (MEFT) (Lu et al., 2000). SCFA signalling pathways are summarised in Figure 1. Additionally, HDACs 4 and 5 contribute to denervation atrophy via activation of atrogenes (Moresi et al., 2010). Furthermore, HDAC inhibitors have been shown to reduce fibrosis and improve muscle function in *mdx* mouse models of Duchenne muscular dystrophy (DMD) (Consalvi et al., 2013). Butyrate also promotes PGC‐1α gene expression, which in one study led to a shift in skeletal muscle fibres from glycolytic to mitochondria‐rich oxidative in mouse models (Gao et al., 2009). Additionally, the same study found that butyrate administration prevented insulin resistance when mice were exposed to a high fat diet, by stimulating thermogenesis and fatty acid oxidation in skeletal muscle and brown adipose tissue mitochondria (Gao et al., 2009).

While not as much is known about interactions with propionate, it does serve several metabolic purposes. Propionate increases insulin‐independent glucose uptake in C2C12 myotubes via activation of GPR‐41 receptors (Han et al., 2014). Propionate can also enter the TCA cycle as succinyl‐CoA where it is converted into oxaloacetate, contributing to hepatic gluconeogenesis (den Besten et al., 2013). Interestingly, certain microbes can convert exercise‐induced lactate into propionate via the methylmalonyl‐CoA pathway, which translated to increased athletic performance in one study in mice (Scheiman et al., 2019).

While the exact mechanism remains elusive, SCFA uptake has been shown to promote insulin‐like growth factor 1 (IGF‐1) production in both the liver and adipose tissue (Yan, Herzog et al., 2016). IGF‐1 is a primary anabolic hormone. Chronic subclinical inflammation (i.e., overexpression of IL‐6) can result in the down‐regulation of IGF‐1 (Maggio et al., 2013), contributing to anabolic resistance in myocytes (Haran et al., 2012). SCFAs also upregulate the NAD‐dependent deacetylase sirtuin‐1 (SIRT1) receptor (Ticinesi et al., 2017). SIRT1 is a redox‐sensitive energy sensor that can positively modulate mitochondrial biogenesis via PGC‐1α deacetylation (Clark & Mach, 2017).

### 2.2 Bile acid signalling

SIRT1 is also a target for secondary bile acids. Primary bile acids are cholesterol derivatives synthesised by hepatocytes in the liver where they are further conjugated with glycine or taurine and then secreted in bile into the small intestine (Swann et al., 2011). Within the small intestine, bile acids regulate bacterial proliferation and overgrowth, while also undergoing deconjugation, dehydrogenation, dehydroxylation and sulfation reactions to become secondary bile acids (Midtvedt, 1974). Because the microbiota plays an integral role in the production of secondary bile acids, microbial diversity also confers diversity on bile acid profile (Swann et al., 2011). Bile acids primarily function to absorb dietary fats and lipid‐soluble vitamins in the small intestine and maintain hepatic cholesterol homeostasis (Swann et al., 2011). Additionally, secondary bile acids have endocrine functions, enabling interaction with mitochondria via binding to the farnesoid X receptor (FXR) and the plasma membrane‐bound bile acid receptor (TGR5) (Swann et al., 2011).

Activation of the FXR reduces insulin resistance and protects against muscle fat deposition (Cipriani et al., 2010). FXR activation also downregulates steroid response element binding protein‐1c (SREBP‐1c), carbohydrate response element binding protein (ChREBP) and peroxisome proliferator‐activated receptor α (PPAR‐α), all of which are found in skeletal muscle playing roles in fatty acid synthesis (Joyce & Gahan, 2016), muscle fibre type determination (Hanke et al., 2011) and uptake and oxidation of fatty acids (Joyce & Gahan, 2016), respectively. Bile acid signalling pathways are summarised in Figure 1. A study using antibiotic deletion of gut microbes observed a disruption of microbial bile acid metabolism, resulting in an increase in TβMCA, a known antagonist of FXR receptors. The disruption of FXR signalling leads to atrophy via disruption of fibroblast growth factor 19 (FGF19; FGF15 in rodents) signalling and downstream extracellular signal‐regulated protein kinase (ERK) pathways (Qiu et al., 2021).

Additionally, secondary bile acids can increase energy expenditure in skeletal muscle cells via interactions with TGR5, resulting in intracellular thyroid hormone activation (Watanabe et al., 2006). One experiment observed during exercise that the unfolded protein response, required for maintenance of endoplasmic reticulum homeostasis during exercise, increased the expression of TGR5, promoting muscle cell differentiation and muscle hypertrophy (Sasaki et al., 2018). TGR5 activation is also associated with lowered pro‐inflammatory cytokine levels (i.e., IL‐1α, IL‐1β, IL‐6 and tumour necrosis factor‐α; Duboc et al., 2014). Activation of TGR5 also suppresses nuclear kappa light‐chain enhancer of activated B cells (NF‐κB) transcriptional activity. NF‐κB induces pro‐inflammatory pathways and is typically tightly controlled. Chronic activation of NF‐κB is associated with inflammation, auto‐immune diseases (Wang et al., 2011) and muscle wasting (Cai et al., 2004).

We are just beginning to fully understand the extent of the inter‐relationship between skeletal muscle and microbial metabolic signalling. Due to the complexity of the underlying physiology, it is evident that there are multiple mechanisms augmenting skeletal muscle development and maintenance. The importance of the interplay between gut microbiota and muscle is evident but remains enigmatic and difficult to unravel. It is apparent that the bidirectionality of the gut–muscle axis creates a positive perpetual loop where exercise and healthy eating (i.e., nutrient supply) contribute to a beneficial diverse commensal population, which in turn promotes the development of stronger muscles permitting increased physical capacity. The emerging evidence strongly points to the requirement of a healthy gut for healthy muscles and whole‐body health.

## 3 THE MUSCULAR DYSTROPHIES

The muscular dystrophies are a set of monogenic disorders characterised by defects in muscle proteins, which contribute to progressive skeletal muscle wasting and weakness (Emery, 2002). Ultimately, individuals with a severe form of muscular dystrophy die prematurely of either respiratory or cardiac failure (Lo Mauro & Aliverti, 2016).

DMD is characterised by mutations in *DMD*, the gene encoding dystrophin, which leads to premature truncation of protein translation, resulting in unstable and non‐functioning dystrophin (Duan et al., 2021). Dystrophin isoforms are expressed ubiquitously throughout the body (e.g., cortical neurons, cerebellar Purkinje cells, the retina, central nervous system, kidney, peripheral nerves, Schwann cells and muscle; Duan et al., 2021). Mutations in *DMD* can also cause a milder disease, Becker muscular dystrophy (BMD), characterised by a later onset and slower, less severe progression. There are thousands of different mutations found in people with DMD and BMD (Duan et al., 2021).

Within muscle, dystrophin links myocyte cytoskeletal F‐actin with the cellular membrane (sarcolemma) via its N‐terminal and C‐terminal domains (Ibraghimov‐Beskrovnaya et al., 1992). Dystrophin is an integral part of a dystrophin‐glycoprotein complex (DGC) that bridges the myocyte cytoskeleton to the extracellular matrix, stabilizing the sarcolemma, protecting the myocyte from contraction‐induced damage and necrosis (Gao & McNally, 2015). Therefore, dystrophin deficiency leads to a disassembly of the DGC, permitting contractile damage with deleterious consequences for muscle cell function.

DMD affects tissues other than skeletal muscle since dystrophin is expressed ubiquitously throughout the body. DMD is also associated with gastrointestinal dysfunction resulting in life‐threatening constipation and metabolic acidosis; these disturbances can further lead to insufficient fluid and caloric intake (lo Cascio et al., 2016), which may contribute to gut microbial dysbiosis, further contributing to the dystrophic pathology.

## 4 MECHANISMS OF MUSCLE WASTING

There are several pathways that contribute to myocyte necrosis in DMD. One major pathway is sarcolemmal weakening. Repeated cycles of contraction generate force and stress on the sarcolemma which is mitigated by the DGC in healthy myocytes. In DMD, the more heavily worked muscles (e.g., diaphragm or heart) are affected earlier, which is why individuals with DMD typically die from respiratory and/or cardiac failure (Duan et al., 2021). Dystrophin also functions to anchor neuronal nitric oxide synthase (nNOS) to the sarcolemma as part of the DGC (Sander et al., 2000). nNOS elicits localised vasodilatation via the release of nitric oxide into the vasculature, blunting sympathetically induced vasoconstriction, allowing for adequate perfusion to exercising muscle beds (Sander et al., 2000). In DMD, nNOS is delocalised to the cytosol, resulting in impaired microcirculation and functional ischaemia (Sander et al., 2000).

Free‐radical damage is also considerably higher within DMD models when compared to normal. In DMD, the microtubule lattice is denser and disorganised, increasing the amounts of stretching and activation of NADPH oxidase 2 (NOX2), producing elevated levels of ROS (Khairallah et al., 2012). Free‐radical levels are also elevated via infiltration of inflammatory cells and dysfunctional mitochondria (Duan et al., 2021). Moreover, levels of glutathione, a vital protective antioxidant, are greatly reduced in DMD, reducing the capacity of muscle to cope with the rising levels of oxidative stress (Duan et al., 2021). Additionally, delocalisation of nNOS into the cytosol results in elevated RNS, which has further deleterious downstream effects (Li et al., 2011).

Calcium is released during muscle contraction from the sarcoplasmic reticulum via calcium release channels/ryanodine receptors (RyR1). Nitrosylation of RyR1 via nitric oxide prevents the binding of the stabilizing protein calstabin resulting in calcium leakage (Bellinger et al., 2009). Calcium overloading can lead to mitochondrial dysfunction and activation of several degradation pathways (e.g., calcium‐dependent calpain protease, phospholipase A2 (PLA2) and mitochondria‐dependent necrosis (Duan et al., 2021)).

Muscle is in a perpetual cycle of breakdown and regeneration. Regeneration is facilitated by the asymmetric division of satellite cells and interactions of DGC proteins. However, in DMD muscle, regeneration is exhausted resulting in muscle wasting, fibrosis and fat replacement (Duan et al., 2021). DMD models with compromised DGCs experience decreased regenerative capacity via altered epigenetic‐mediated gene transcription. The mitogen‐activated protein kinase (MAPK) p38γ is regulated via interactions with the DGC during stem cell divisions. The p38γ pathway phosphorylates the cofactor Carm1 preventing it from binding to Pax7 and promoting the expression of *Myf5*, a key marker in muscle stem cell differentiation (Chang et al., 2018). Regenerative potential is also blunted in DMD models due to matrix restructuring and chronic inflammation (Duan et al., 2021). Chronic inflammation increases transforming growth factor β1 (TGF‐β1) levels, which leads to continuous connective tissue remodelling and eventually to fibrosis (Rosenberg et al., 2015).

Interestingly, Carm1 is also implicated in the positive regulation of autophagy, functioning as a nuclear transcriptional co‐activator to induce genes necessary for autophagy (Shin et al., 2016). In normal muscle, autophagy is responsible for the maintenance of the cellular environment removing defective organelles and protein aggregates. However, in DMD models, autophagy is blunted. Additionally, beyond Carm1, NOX2‐induced oxidative stress can impair autophagy via the activation of the autophagy repressor mammalian target of rapamycin through the phosphoinositide 3‐kinase/Akt pathway (Pal et al., 2014). Accumulation of defective organelles and dysfunctional proteins contributes to muscle degeneration (Duan et al., 2021).

The constant cycle of degeneration and repair, paired with insufficient stem cell‐dependent regeneration, leads to a replacement of tissue, which inevitably leads to decreased functionality and cardiorespiratory failure. The recovery potential of muscle is limited and in the context of DMD, satellite cell proliferation cannot match the constant breakdown; this leads to muscular necrosis without replacement, inflammation, fibrotic remodelling and eventually adipose replacement (Duan et al., 2021; Gao & McNally, 2015; Klingler et al., 2012; Mhandire et al., 2022). In humans, remodelling typically occurs in the posterior calf muscle first (Klingler et al., 2012); however, by 10–12 years of age individuals begin to experience progressive respiratory dysfunction due to fibrotic changes in the respiratory muscles (Passamano et al., 2012). As respiratory muscle weakness progresses, maximal inspiratory and expiratory pressures decrease, reducing vital capacity, eventually leading to respiratory instability and insufficiency (Duan et al., 2021; Mhandire et al., 2022).

## 5 GUT MICROBIOTA‐DIRECTED THERAPIES FOR RESPIRATORY MUSCULAR DYSFUNCTION

The gut microbiota can be thought of as a transducer of nutrient signals for the host, with the ability to generate pro‐anabolic signals and produce mediators that regulate metabolic homeostasis, insulin sensitivity and inflammation (Ticinesi et al., 2017). Both manipulation of the gut microbial population and introduction of specific substrates can beneficially alter skeletal muscle, potentially alleviating DMD‐associated pathologies.

Altering the host microbial profile has shown promising steps in enhancing and/or preserving skeletal muscle. In one study, muscle properties were successfully transferred from a pig to germ‐free mice via faecal microbiota transplantation. Following transplantation, the germ‐free mice exhibited the same higher body mass, skeletal muscle fibre characteristics and lipid metabolism as their donors (Yan, Diao et al., 2016). In a separate study, transplantation from specific pathogen‐free mice to germ‐free mice resulted in an increase in skeletal muscle mass, reduction in muscle atrophy markers, improved oxidative metabolic capacity of the muscle and elevated expression of neuromuscular junction assembly genes (Lahiri et al., 2019). Additionally, supplementation of specific microbial strains has also conferred beneficial changes to hosts. *Lactobacillus plantarum* supplementation has shown positive effects on muscle mass and function in both young and aged mice, and young and older adult humans (Chen et al., 2016; Huang et al., 2019; Lee, Chen, et al., 2022; Lee, Liao, et al., 2021; Lee, Liao, et al., 2022; Lee, Tu, et al., 2021). Oral supplementation of specific types of lactobacilli has also been shown to mitigate muscle wasting in leukaemia mouse models, reducing atrophy marker expression and inflammation (Bindels et al., 2012), which may translate to muscular dystrophy.

**FIGURE 2:**
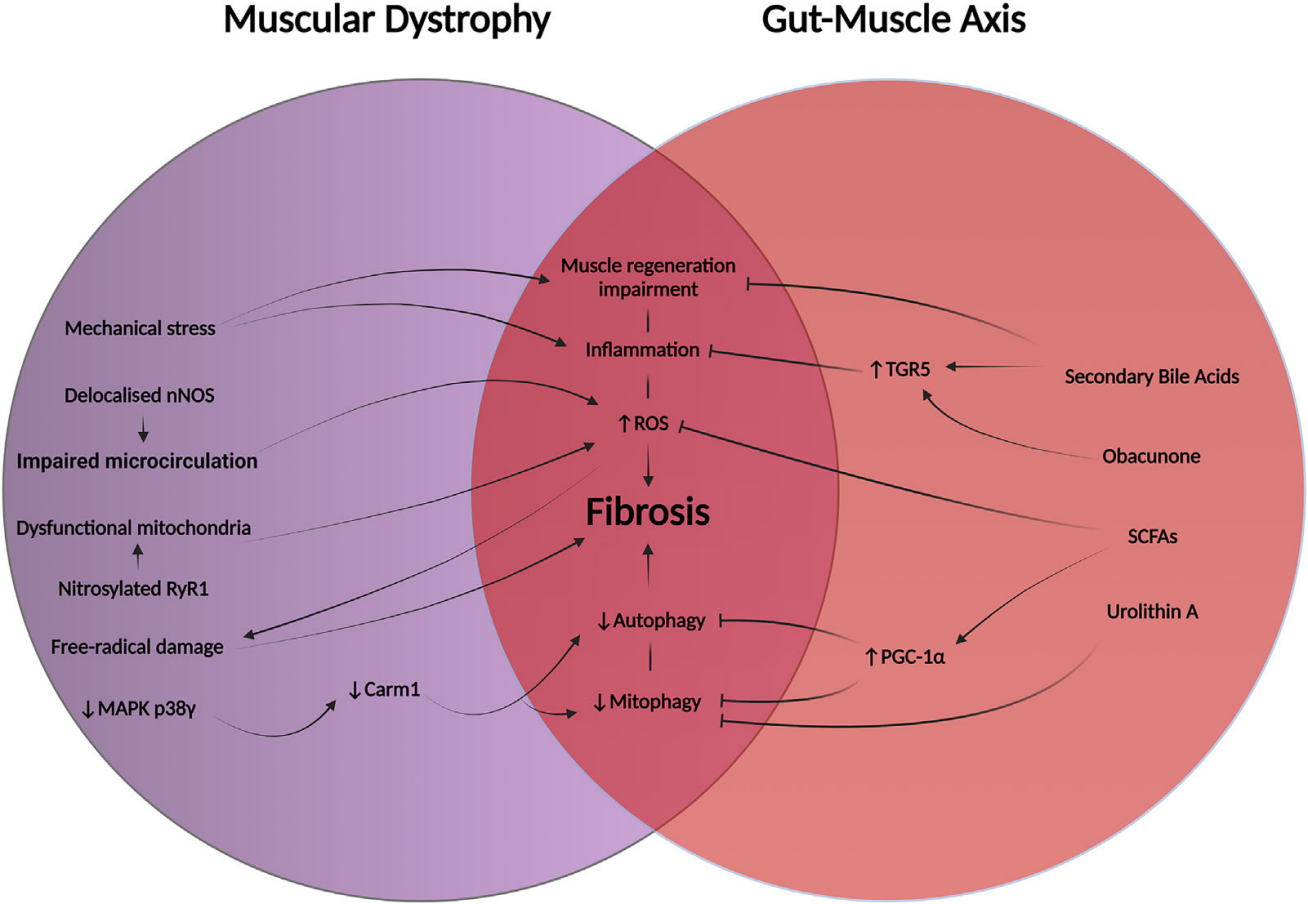
Diagram of the interplay between muscular dystrophy and gut–muscle axis signalling. Supplementation and microbial signalling can offset several of the dystrophic pathways that contribute to fibrosis.

Studies investigating how microbial metabolites affect skeletal muscle have also yielded promising results. Supplementation with SCFA has been shown to improve muscle mass (Walsh et al., 2015) and exercise capacity (Okamoto et al., 2019) within old and antibiotic‐treated mouse models, respectively; however, the extent of the treatment is limited. In one study, SCFAs prevented atrophy and increased muscular strength in germ‐free mice but could not completely rescue the muscle phenotype (Lahiri et al., 2019). Microbial interactions within the host may engage multiple pathways, suggesting that a more holistic approach may be necessary in a pathology as complex as muscular dystrophy. Additionally, metabolites activating the G‐protein‐coupled bile acid receptor Gpbar1 (TGR5) have also been shown to improve skeletal muscle function in mice. The interplay between mechanisms of muscular dystrophy and microbial signalling is illustrated in Figure 2. One study observed that the dietary supplement obacunone (found in citrus) stimulated muscle hypertrophy and prevented obesity and hyperglycaemia via the activation of TGR5 and PPAR‐γ (Horiba et al., 2015). This makes obacunone a potential therapeutic option; however, in addition, TGR5 can be stimulated via bile acid signalling (Swann et al., 2011) and PPAR‐γ is stimulated by butyrate (Byndloss et al., 2017), both naturally produced microbial metabolites.

Another potential therapeutic approach is to augment autophagy and the clearance of dysfunctional mitochondria (mitophagy). Inadequate autophagy can contribute to muscle wasting. Urolithin A, produced naturally in the colon by gut bacteria from ellagitannins and ellagic acid derived from pomegranate, berries and nuts, has shown promise (D'Amico et al., 2021). One study observed that urolithin A induces mitophagy, preventing the accumulation of dysfunctional mitochondria and extended lifespan in *Caenorhabditis elegans* models. Additionally, urolithin A improved exercise capacity in aged mouse models and young rats (Ryu et al., 2016). Urolithin A not only recovered mitophagy, but also improved regenerative ability of muscle stem cells and increased skeletal muscle respiratory capacity, increasing survival of dystrophic mice (Luan et al., 2021).

**FIGURE 3:**
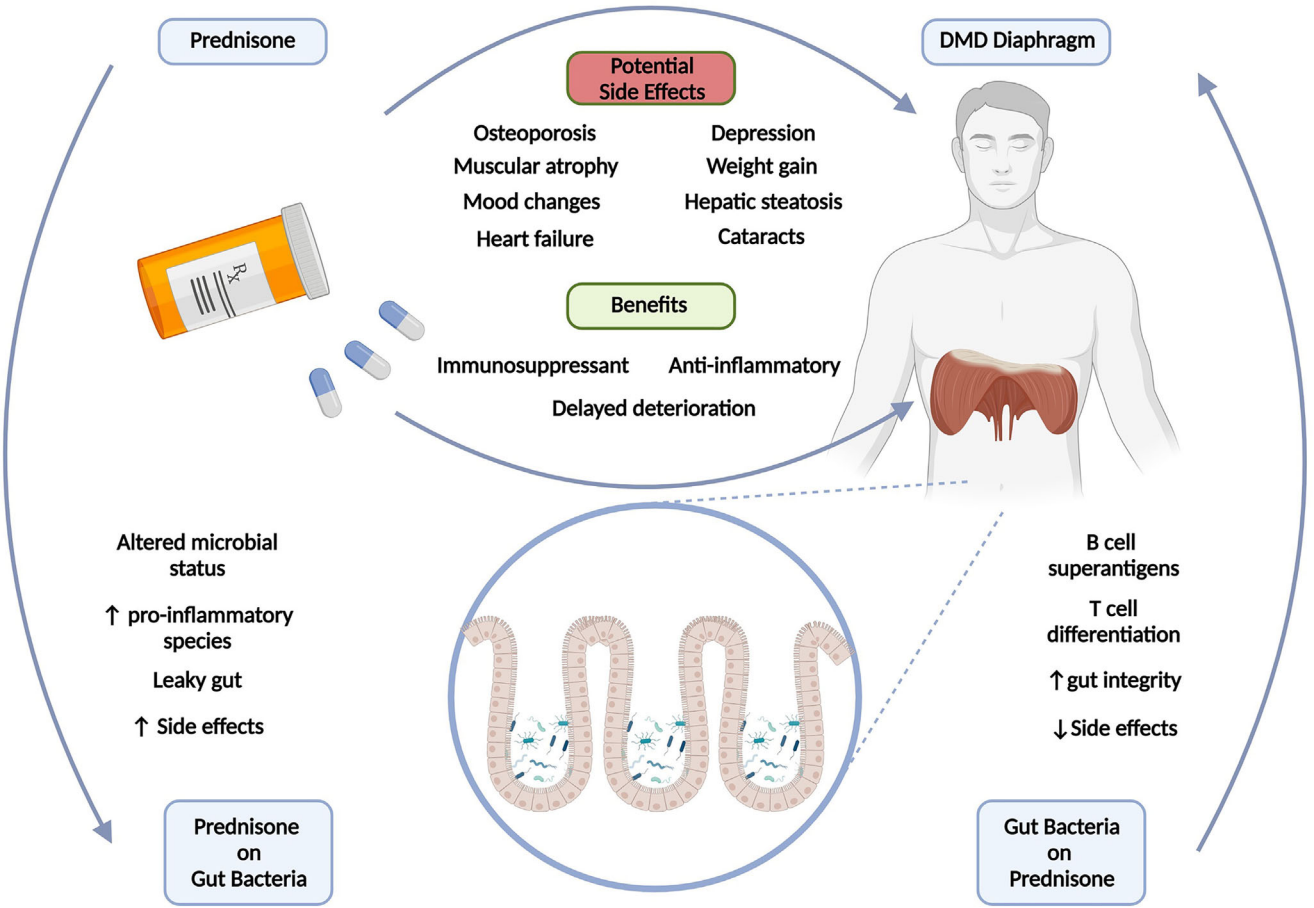
Interactions between prednisone, muscle and the gut. Prednisone is the gold standard in muscular dystrophy treatment, but it is associated with many side effects. Prednisone also affects gut microbial status. Positively altering the gut microbiome can increase the efficacy of prednisone while offsetting some of the associated side effects.

Currently glucocorticoids (i.e., prednisone and prednisolone) are the gold standard treatment in DMD (Gloss et al., 2016). Glucocorticoids are anti‐inflammatory and immunosuppressing therapeutic drugs which function to mitigate muscle wasting in individuals with DMD. However, glucocorticoid use is only a temporary therapeutic solution that merely delays the inevitable degeneration and cardiorespiratory failure while introducing myriad problems. Long term use of glucocorticoids can induce deleterious effects on numerous systems (e.g., osteoporosis, muscular atrophy, weight gain, hepatic steatosis, mood changes, depression, cataracts and more (Oray et al., 2016). Glucocorticoid use also alters gut microbial status, which is implicated in the development of side effects. A study using lupus mouse models observed a significant change in microbial populations after prednisone administration. The authors postulated that the shift in microbial status enhanced the therapeutic efficacy of prednisone (Wang et al., 2021). Prednisone treatment resulted in a shift in phyla, favouring pro‐inflammatory bacteria while conversely favouring bacteria capable of producing B cell superantigens and regulating T cell differentiation, boosting the efficacy of prednisone (Table 1). Interestingly, faecal microbial transplantation from mice exposed to prednisone alleviated the lupus, further suggesting that a beneficial change to microbial status contributed to the therapeutic effect of prednisone (Wang et al., 2021). Similarly, in another study, glucocorticoid driven osteoporosis was ameliorated by supplementation with *Lactobacillus reuteri*, a bacterium associated with the protection of intestinal epithelial barrier integrity (Schepper et al., 2019). To confirm that a leaky gut contributed to the osteoporosis, a mucus supplement was used which yielded similar results to a probiotic strategy (Schepper et al., 2019). These findings indicate a direct relationship between the gut microbiome and the efficacy and side effects associated with glucocorticoid therapy (Figure 3).

**TABLE 1:** Microbial products and sensitivity to prednisone and antibiotics. Brief overview of the key bacterial genera responsible for short chain fatty acid and secondary bile acid synthesis, and their sensitivity to prednisone and various representative antibiotics.

Genus	Short chain fatty acid production	Secondary bile acid synthesis	Prednisone sensitivity	Antibiotic sensitivity
*Prevotella*	Acetate			Metronidazole, vancomycin
*Ruminococcus*	Acetate, butyrate, propionate		Susceptible	Doxycycline, metronidazole, vancomycin
*Bifidobacterium*	Acetate, propionate	Hydrolysis capable		Amoxicillin, vancomycin
*Bacteroides*	Acetate, propionate	Hydrolysis capable	Susceptible	Amoxicillin, metronidazole, vancomycin
*Clostridium*	Acetate, propionate	Hydrolysis capable	Susceptible	Metronidazole, nitrofurantoin, vancomycin
*Streptococcus*	Acetate		Susceptible	Amoxicillin, clarithromycin
*Akkermansia*	Acetate, butyrate, propionate		Susceptible	Doxycycline
*Coprococcus*	Acetate, propionate			Metronidazole, vancomycin
*Fusobacterium*	Acetate			Amoxicillin, metronidazole
*Eubacterium*	Acetate, butyrate		Susceptible	Amoxicillin, clarithromycin
*Dialister*	Acetate			Amoxicillin, doxycycline, vancomycin
*Oxalobacter*	Acetate			Clarithromycin, doxycycline, metronidazole
*Enterococcus*	Acetate	Hydrolysis capable		Nitrofurantoin
*Lactobacillus*	Acetate, propionate	Hydrolysis capable	Susceptible	Doxycycline
*Roseburia*	Acetate, butyrate, propionate			Amoxicillin, doxycycline, vancomycin
*Faecalibacterium*	Acetate, butyrate			Doxycycline, metronidazole, vancomycin

## 6 CONCLUSION

The bidirectional gut–muscle axis allows for manipulation of both the gut environment and muscle development. Importantly, exercise and use of muscles can positively alter gut microbial populations as well as improve overall health; however, in the context of DMD, exercise can cause undue stress on vital muscles resulting in quicker decline. Most of the current literature revolving around the gut–muscle axis is primarily focused on animal models; however, a disruption of gut microbial status is evident in athletic humans and those that experience other forms of muscle wasting such as sarcopenia and cachexia, making the gut microbiome a biomarker of human health. Growing evidence points to the potential for a combined pro‐ and prebiotic regimen designed to populate the gut with an optimal microbial profile and complementary provision of optimal substrates to alleviate muscle wasting via positive modulation of the gut microbiota. While it is too soon to tell whether the adverse phenotype in muscular dystrophies can be ameliorated, it is promising to consider that, based on animal studies, microbe‐based therapeutics could potentially translate to humans and mitigate muscle wasting and prolong life expectancy, particularly when paired with other established interventional strategies such as prednisone treatment and exercise.

## AUTHOR CONTRIBUTIONS

All authors have read and approved the final version of this manuscript and agree to be accountable for all aspects of the work in ensuring that questions related to the accuracy or integrity of any part of the work are appropriately investigated and resolved. All persons designated as authors qualify for authorship, and all those who qualify for authorship are listed.

## CONFLICT OF INTEREST

The authors declare no conflicts of interest.

## References

[eph13390-bib-0001] Bäckhed, F. , Ding, H. , Wang, T. , Hooper, L. V. , Young Koh, G. , Nagy, A. , Semenkovich, C. F. , & Gordon, J. I. (2004). The gut microbiota as an environmental factor that regulates fat storage. Proceedings of the National Academy of Sciences, USA, 101(44), 15718–15723. 10.1073/pnas.0407076101 PMC524219 15505215

[eph13390-bib-0002] Bäckhed, F. , Manchester, J. K. , Semenkovich, C. F. , & Gordon, J. I. (2007). Mechanisms underlying the resistance to diet‐induced obesity in germ‐free mice. Proceedings of the National Academy of Sciences, USA, 104(3), 979–984. 10.1073/pnas.0605374104 PMC1764762 17210919

[eph13390-bib-0003] Barton, W. , Penney, N. C. , Cronin, O. , Garcia‐Perez, I. , Molloy, M. G. , Holmes, E. , Shanahan, F. , Cotter, P. D. , & O'Sullivan, O. (2018). The microbiome of professional athletes differs from that of more sedentary subjects in composition and particularly at the functional metabolic level. Gut, 67(4), 625–633. 28360096 10.1136/gutjnl-2016-313627

[eph13390-bib-0004] Bellinger, A. M. , Reiken, S. , Carlson, C. , Mongillo, M. , Liu, X. , Rothman, L. , Matecki, S. , Lacampagne, A. , & Marks, A. R. (2009). Hypernitrosylated ryanodine receptor calcium release channels are leaky in dystrophic muscle. Nature Medicine, 15(3), 325–330. 10.1038/nm.1916 PMC2910579 19198614

[eph13390-bib-0005] Bindels, L. B. , Beck, R. , Schakman, O. , Martin, J. C. , de Backer, F. , Sohet, F. M. , Dewulf, E. M. , Pachikian, B. D. , Neyrinck, A. M. , Thissen, J. P. , Verrax, J. , Calderon, P. B. , Pot, B. , Grangette, C. , Cani, P. D. , Scott, K. P. , & Delzenne, N. M. (2012). Restoring specific lactobacilli levels decreases inflammation and muscle atrophy markers in an acute leukemia mouse model. PLoS ONE, 7(6), e37971. 22761662 10.1371/journal.pone.0037971 PMC3384645

[eph13390-bib-0006] Bindels, L. B. , & Delzenne, N. M. (2013). Muscle wasting: The gut microbiota as a new therapeutic target? International Journal of Biochemistry and Cell Biology, 45(10), 2186–2190. 23831839 10.1016/j.biocel.2013.06.021

[eph13390-bib-0007] Byndloss, M. X. , Olsan, E. E. , Rivera‐Chávez, F. , Tiffany, C. R. , Cevallos, S. A. , Lokken, K. L. , Torres, T. P. , Byndloss, A. J. , Faber, F. , Gao, Y. , Litvak, Y. , Lopez, C. A. , Xu, G. , Napoli, E. , Giulivi, C. , Tsolis, R. M. , Revzin, A. , Lebrilla, C. , & Bäumler, A. J. (2017). Microbiota‐activated PPAR‐γ‐signaling inhibits dysbiotic Enterobacteriaceae expansion HHS Public Access. Science, 357(6351), 570–575. 28798125 10.1126/science.aam9949 PMC5642957

[eph13390-bib-0008] Cai, D. , Daniel Frantz, J. , Tawa, N. E. , Melendez, P. A. , Oh, B.‐C. , Lidov, H. G. W. , Hasselgren, P.‐O. , Frontera, W. R. , Lee, J. , Glass, D. J. , & Shoelson, S. E. (2004). IKK/NF‐B activation causes severe muscle wasting in mice. Cell, 119(2), 285–298. 15479644 10.1016/j.cell.2004.09.027

[eph13390-bib-0009] Chang, N. C. , Sincennes, M. C. , Chevalier, F. P. , Brun, C. E. , Lacaria, M. , Segalés, J. , Muñoz‐Cánoves, P. , Ming, H. , & Rudnicki, M. A. (2018). The dystrophin glycoprotein complex regulates the epigenetic activation of muscle stem cell commitment. Cell Stem Cell, 22(5), 755–768.e6. 29681515 10.1016/j.stem.2018.03.022 PMC5935555

[eph13390-bib-0010] Chen, Y. M. , Wei, L. , Chiu, Y. S. , Hsu, Y. J. , Tsai, T. Y. , Wang, M. F. , & Huang, C. C. (2016). *Lactobacillus plantarum* TWK10 supplementation improves exercise performance and increases muscle mass in mice. Nutrients, 8(4), 205. 27070637 10.3390/nu8040205 PMC4848674

[eph13390-bib-0011] Cipriani, S. , Mencarelli, A. , Palladino, G. , & Fiorucci, S. (2010). FXR activation reverses insulin resistance and lipid abnormalities and protects against liver steatosis in Zucker (fa/fa) obese rats. Journal of Lipid Research, 51(4), 771–784. 19783811 10.1194/jlr.M001602 PMC2842143

[eph13390-bib-0012] Claesson, M. J. , Jeffery, I. B. , Conde, S. , Power, S. E. , O'Connor, E. M. , Cusack, S. , Harris, H. M. , Coakley, M. , Lakshminarayanan, B. , O'Sullivan, O. , Fitzgerald, G. F. , Deane, J. , O'Connor, M. , Harnedy, N. , O'Connor, K. , O'Mahony, D. , van Sinderen, D. , Wallace, M. , Brennan, L. , … O'Toole, P. W. (2012). Gut microbiota composition correlates with diet and health in the elderly. Nature, 488(7410), 178–184. 22797518 10.1038/nature11319

[eph13390-bib-0013] Clark, A. , & Mach, N. (2017). The crosstalk between the gut microbiota and mitochondria during exercise. Frontiers in Physiology, 8, 319. 28579962 10.3389/fphys.2017.00319 PMC5437217

[eph13390-bib-0014] Clarke, S. F. , Murphy, E. F. , Lucey, A. J. , Humphreys, M. , Hogan, A. , Hayes, P. , Jeffery, I. B. , Wood‐Martin, R. , Kerins, D. M. , Quigley, E. , Paul Ross, R. , O'Toole, P. W. , Molloy, M. G. , Falvey, E. , Shanahan, F. , Cotter, P. D. , & Fergus Shanahan, P. (2014). Exercise and associated dietary extremes impact on gut microbial diversity. Gut, 63(12), 1913–1920. 25021423 10.1136/gutjnl-2013-306541

[eph13390-bib-0015] Consalvi, S. , Mozzetta, C. , Bettica, P. , Germani, M. , Fiorentini, F. , del Bene, F. , Rocchetti, M. , Leoni, F. , Monzani, V. , Mascagni, P. , Lorenzo Puri, P. , & Saccone, V. (2013). Preclinical studies in the mdx mouse model of Duchenne muscular dystrophy with the histone deacetylase inhibitor givinostat. Molecular Medicine, 19(1), 79–87. 23552722 10.2119/molmed.2013.00011 PMC3667212

[eph13390-bib-0016] D'Amico, D. , Andreux, P. A. , Valdés, P. , Singh, A. , Rinsch, C. , & Auwerx, J. (2021). Impact of the natural compound Urolithin A on health, disease, and aging. Trends in Molecular Medicine, 27(7), 687–699. 34030963 10.1016/j.molmed.2021.04.009

[eph13390-bib-0017] den Besten, G. , Lange, K. , Havinga, R. , van Dijk, T. H. , Gerding, A. , van Eunen, K. , Müller, M. , Groen, A. K. , Hooiveld, G. J. , Bakker, B. M. , Reijngoud, D.‐J. , den Besten, G. , van Dijk, T. , & van Eunen, K. (2013). Gut‐derived short‐chain fatty acids are vividly assimilated into host carbohydrates and lipids. American Journal of Physiology. Gastrointestinal and Liver Physiology, 305(12), G900–G910. 24136789 10.1152/ajpgi.00265.2013

[eph13390-bib-0018] Donohoe, D. R. , Garge, N. , Zhang, X. , Sun, W. , O'Connell, T. M. , Bunger, M. K. , & Bultman, S. J. (2011). The microbiome and butyrate regulate energy metabolism and autophagy in the mammalian colon. Cell Metabolism, 13(5), 517–526. 21531334 10.1016/j.cmet.2011.02.018 PMC3099420

[eph13390-bib-0019] Duan, D. , Goemans, N. , Takeda, S. , Mercuri, E. , & Aartsma‐Rus, A. (2021). Duchenne muscular dystrophy. Nature Reviews Disease Primers, 7(1), 13. 10.1038/s41572-021-00248-3 PMC10557455 33602943

[eph13390-bib-0020] Duboc, H. , Taché, Y. , & Hofmann, A. F. (2014). The bile acid TGR5 membrane receptor: From basic research to clinical application. Digestive and Liver Disease, 46(4), 302–312. 24411485 10.1016/j.dld.2013.10.021 PMC5953190

[eph13390-bib-0021] Ellingsgaard, H. , Hauselmann, I. , Schuler, B. , Habib, A. M. , Baggio, L. L. , Meier, D. T. , Eppler, E. , Bouzakri, K. , Wueest, S. , Muller, Y. D. , Hansen, A. M. , Reinecke, M. , Konrad, D. , Gassmann, M. , Reimann, F. , Halban, P. A. , Gromada, J. , Drucker, D. J. , Gribble, F. M. , … Donath, M. Y. (2011). Interleukin‐6 enhances insulin secretion by increasing glucagon‐like peptide‐1 secretion from L cells and alpha cells. Nature Medicine, 17(11), 1481–1489. 10.1038/nm.2513 PMC4286294 22037645

[eph13390-bib-0022] Emery, A. E. H. (2002). The muscular dystrophies. Lancet, 359(9307), 687–695. 11879882 10.1016/S0140-6736(02)07815-7

[eph13390-bib-0023] Francisco, G. , & Malagelada, J. R. (2003). Gut flora in health and disease. Lancet, 361(9356), 512–519. 12583961 10.1016/S0140-6736(03)12489-0

[eph13390-bib-0024] Furber, M. J. W. , Young, G. R. , Holt, G. S. , Pyle, S. , Davison, G. , Roberts, M. G. , Roberts, J. D. , Howatson, G. , & Smith, D. L. (2022). Gut microbial stability is associated with greater endurance performance in athletes undertaking dietary periodization. mSystems, 7(3), e0012922. 35579384 10.1128/msystems.00129-22 PMC9238380

[eph13390-bib-0025] Gao, Q. Q. , & McNally, E. M. (2015). The dystrophin complex: Structure, function, and implications for therapy. Comprehensive Physiology, 5(3), 1223–1239. 26140716 10.1002/cphy.c140048 PMC4767260

[eph13390-bib-0026] Gao, Z. , Yin, J. , Zhang, J. , Ward, R. E. , Martin, R. J. , Lefevre, M. , Cefalu, W. T. , & Ye, J. (2009). Butyrate improves insulin sensitivity and increases energy expenditure in mice. Diabetes, 58(7), 1509–1517. 19366864 10.2337/db08-1637 PMC2699871

[eph13390-bib-0027] Gloss, D. , Moxley Iii, R. T. , Ashwal, S. , & Oskoui, M. (2016). Practice guideline update summary: Corticosteroid treatment of Duchenne muscular dystrophy. Neurology, 86(5), 465–472. 26833937 10.1212/WNL.0000000000002337 PMC4773944

[eph13390-bib-0028] Goffredo, M. , Mass, K. , Parks, E. J. , Wagner, D. A. , McClure, E. A. , Graf, J. , Savoye, M. , Pierpont, B. , Cline, G. , & Santoro, N. (2016). Role of gut microbiota and short chain fatty acids in modulating energy harvest and fat partitioning in youth. Journal of Clinical Endocrinology and Metabolism, 101(11), 4367–4376. 27648960 10.1210/jc.2016-1797 PMC5095239

[eph13390-bib-0029] Hakozaki, T. , Nolin‐Lapalme, A. , Kogawa, M. , Okuma, Y. , Nakamura, S. , Moreau‐Amaru, D. , Tamura, T. , Hosomi, Y. , Takeyama, H. , Richard, C. , Hosokawa, M. , & Routy, B. (2022). Cancer cachexia among patients with advanced non‐small‐cell lung cancer on immunotherapy: An observational study with exploratory gut microbiota analysis. Cancers, 14(21), 5405. 36358821 10.3390/cancers14215405 PMC9658074

[eph13390-bib-0030] Han, J.‐H. , Kim, I.‐S. , Jung, S.‐H. , Lee, S.‐G. , Son, H.‐Y. , & Myung, C.‐S. (2014). The effects of propionate and valerate on insulin responsiveness for glucose uptake in 3T3‐L1 adipocytes and C2C12 myotubes via G protein‐coupled receptor 41. PLoS ONE, 9(4), e95268. 24748202 10.1371/journal.pone.0095268 PMC3991595

[eph13390-bib-0031] Hanke, N. , Scheibe, R. J. , Manukjan, G. , Ewers, D. , Umeda, P. K. , Chang, K. C. , Kubis, H. P. , Gros, G. , & Meissner, J. D. (2011). Gene regulation mediating fiber‐type transformation in skeletal muscle cells is partly glucose‐ and ChREBP‐dependent. Biochimica et Biophysica Acta – Molecular Cell Research, 1813(3), 377–389. 10.1016/j.bbamcr.2010.12.021 21215280

[eph13390-bib-0032] Haran, P. H. , Rivas, D. A. , & Fielding, R. A. (2012). Role and potential mechanisms of anabolic resistance in sarcopenia. Journal of Cachexia, Sarcopenia and Muscle, 3(3), 157–162. 22589021 10.1007/s13539-012-0068-4 PMC3424190

[eph13390-bib-0033] Horiba, T. , Katsukawa, M. , Mita, M. , & Sato, R. (2015). Dietary obacunone supplementation stimulates muscle hypertrophy, and suppresses hyperglycemia and obesity through the TGR5 and PPARγ pathway. Biochemical and Biophysical Research Communications, 463(4), 846–852. 26051277 10.1016/j.bbrc.2015.06.022

[eph13390-bib-0034] Huang, W. C. , Lee, M. C. , Lee, C. C. , Ng, K. S. , Hsu, Y. J. , Tsai, T. Y. , Young, S. L. , Lin, J. S. , & Huang, C. C. (2019). Effect of *Lactobacillus plantarum* TWK10 on exercise physiological adaptation, performance, and body composition in healthy humans. Nutrients, 11(11), 2836. 31752370 10.3390/nu11112836 PMC6893516

[eph13390-bib-0035] Ibraghimov‐Beskrovnaya, O. , Ervasti, J. M. , Leveille, C. J. , Slaughter, C. A. , Sernett, S. W. , & Campbell, K. P. (1992). Primary structure of dystrophin‐associated glycoproteins linking dystrophin to the extracellular matrix. Nature, 355(6362), 696–702. 1741056 10.1038/355696a0

[eph13390-bib-0036] Joyce, S. A. , & Gahan, C. G. M. (2016). Bile acid modifications at the Microbe‐host interface: Potential for nutraceutical and pharmaceutical interventions in host health. Annual Review of Food Science and Technology, 7(1), 313–333. 10.1146/annurev-food-041715-033159 26772409

[eph13390-bib-0037] Kang, L. , Li, P. , Wang, D. , Wang, T. , Hao, D. , & Qu, X. (2021). Alterations in intestinal microbiota diversity, composition, and function in patients with sarcopenia. Scientific Reports, 11(1), 4628. 33633246 10.1038/s41598-021-84031-0 PMC7907362

[eph13390-bib-0038] Khairallah, R. J. , Shi, G. , Sbrana, F. , Prosser, B. L. , Borroto, C. , Mazaitis, M. J. , Hoffman, E. P. , Mahurkar, A. , Sachs, F. , Sun, Y. , Chen, Y. W. , Raiteri, R. , Lederer, W. J. , Dorsey, S. G. , & Ward, C. W. (2012). Microtubules underlie dysfunction in Duchenne muscular dystrophy. Science Signaling, 5(236), ra56. 22871609 10.1126/scisignal.2002829 PMC3835660

[eph13390-bib-0039] Klingler, W. , Jurkat‐Rott, K. , Lehmann‐Horn, F. , & Schleip, R. (2012). The role of fibrosis in Duchenne muscular dystrophy. Acta Myologica, 31(3), 184–195. 23620650 PMC3631802

[eph13390-bib-0040] Lahiri, S. , Kim, H. , Garcia‐Perez, I. , Reza, M. , Martin, K. A. , Kundu, P. , Cox, L. M. , Selkrig, J. , Posma, J. M. , Zhang, H. , Padmanabhan, P. , Moret, C. , Gulyás, B. , Blaser, M. J. , Auwerx, J. , Holmes, E. , Nicholson, J. , Wahli, W. , & Pettersson, S. (2019). The gut microbiota influences skeletal muscle mass and function in mice HHS Public Access. Science Translational Medicine, 11(502), eaan5662. 31341063 10.1126/scitranslmed.aan5662 PMC7501733

[eph13390-bib-0041] Lee, C. C. , Liao, Y. C. , Lee, M. C. , Cheng, Y. C. , Chiou, S. Y. , Lin, J. S. , Huang, C. C. , & Watanabe, K. (2022). Different impacts of heat‐killed and viable *Lactiplantibacillus plantarum* TWK10 on exercise performance, fatigue, body composition, and gut microbiota in humans. Microorganisms, 10(11), 2181. 36363775 10.3390/microorganisms10112181 PMC9692508

[eph13390-bib-0042] Lee, C. C. , Liao, Y. C. , Lee, M. C. , Lin, K. J. , Hsu, H. Y. , Chiou, S. Y. , Young, S. L. , Lin, J. S. , Huang, C. C. , & Watanabe, K. (2021). *Lactobacillus plantarum* TWK10 attenuates aging‐associated muscle weakness, bone loss, and cognitive impairment by modulating the gut microbiome in mice. Frontiers in Nutrition, 8, 708096. 34722603 10.3389/fnut.2021.708096 PMC8548577

[eph13390-bib-0043] Lee, M. C. , Chen, M. J. , Huang, H. W. , Wu, W. K. , Lee, Y. W. , Kuo, H. C. , & Huang, C. C. (2022). Probiotic *Lactiplantibacillus plantarum* Tana isolated from an international weightlifter enhances exercise performance and promotes antifatigue effects in mice. Nutrients, 14(16), 3308. 36014816 10.3390/nu14163308 PMC9416726

[eph13390-bib-0044] Lee, M.‐C. , Tu, Y.‐T. , Lee, C.‐C. , Tsai, S.‐C. , Hsu, H.‐Y. , Tsai, T.‐Y. , Liu, T.‐H. , Young, S.‐L. , Lin, J.‐S. , & Huang, C.‐C. (2021). *Lactobacillus plantarum* TWK10 improves muscle mass and functional performance in frail older adults: A randomized, double‐blind clinical trial. Microorganisms, 9(7), 1466. 34361902 10.3390/microorganisms9071466 PMC8305125

[eph13390-bib-0045] Li, D. , Yue, Y. , Lai, Y. , Hakim, C. H. , & Duan, D. (2011). Nitrosative stress elicited by nNOSμ delocalization inhibits muscle force in dystrophin‐null mice. Journal of Pathology, 223(1), 88–98. 21125668 10.1002/path.2799 PMC3109084

[eph13390-bib-0046] lo Cascio, C. M. , Goetze, O. , Latshang, T. D. , Bluemel, S. , Frauenfelder, T. , & Bloch, K. E. (2016). Gastrointestinal dysfunction in patients with Duchenne muscular dystrophy. PLoS ONE, 11(10), e0163779. 27736891 10.1371/journal.pone.0163779 PMC5063332

[eph13390-bib-0047] Lochlainn, M. N. , Bowyer, R. C. E. , & Steves, C. J. (2018). Dietary protein and muscle in aging people: The potential role of the gut microbiome. Nutrients, 10(7), 929. 30036990 10.3390/nu10070929 PMC6073774

[eph13390-bib-0048] Lo Mauro, A. , & Aliverti, A. (2016). Physiology of respiratory disturbances in muscular dystrophies. Breathe, 12(2), 318–327. 28210319 10.1183/20734735.012716 PMC5297947

[eph13390-bib-0049] Lu, J. , Mckinsey, T. A. , Zhang, C.‐L. , & Olson, E. N. (2000). Regulation of skeletal myogenesis by association of the MEF2 transcription factor with Class II histone deacetylases. Molecular Cell, 6(2), 233–244. 10983972 10.1016/s1097-2765(00)00025-3

[eph13390-bib-0050] Luan, P. , Amico, D. D. , Andreux, P. A. , Laurila, P. P. , Wohlwend, M. , Li, H. , Lima, T. I. , Place, N. , Rinsch, C. , Zanou, N. , & Auwerx, J. (2021). Urolithin A improves muscle function by inducing mitophagy in muscular dystrophy. Science Translational Medicine, 13(588), eabb0319. 33827972 10.1126/scitranslmed.abb0319

[eph13390-bib-0051] Mach, N. , & Fuster‐Botella, D. (2017). Endurance exercise and gut microbiota: A review. Journal of Sport and Health Science, 6(2), 179–197. 30356594 10.1016/j.jshs.2016.05.001 PMC6188999

[eph13390-bib-0052] Maggio, M. , de Vita, F. , Lauretani, F. , Buttò, V. , Bondi, G. , Cattabiani, C. , Nouvenne, A. , Meschi, T. , Dall'aglio, E. , & Ceda, G. P. (2013). IGF‐1, the cross road of the nutritional, inflammatory and hormonal pathways to frailty. Nutrients, 5(10), 4184–4205. 24152751 10.3390/nu5104184 PMC3820068

[eph13390-bib-0053] Manickam, R. , Yun, H. , Oh, P. , Tan, C. K. , Paramalingam, E. , & Wahli, W. (2018). Metronidazole causes skeletal muscle atrophy and modulates muscle chronometabolism. International Journal of Molecular Sciences Article, 19(8), 2418. 10.3390/ijms19082418 PMC6121908 30115857

[eph13390-bib-0054] Mhandire, D. Z. , Burns, D. P. , Roger, A. L. , O'Halloran, K. D. , & ElMallah, M. K. (2022). Breathing in Duchenne muscular dystrophy: Translation to therapy. The Journal of Physiology, 600(15), 3465–3482. 35620971 10.1113/JP281671 PMC9357048

[eph13390-bib-0055] Midtvedt, T. (1974). Microbial bile acid transformation. American Journal of Clinical Nutrition, 27(11), 1341–1347. 4217103 10.1093/ajcn/27.11.1341

[eph13390-bib-0056] Moresi, V. , Williams, A. H. , Meadows, E. , Flynn, J. M. , Potthoff, M. J. , McAnally, J. , Shelton, J. M. , Backs, J. , Klein, W. H. , Richardson, J. A. , Bassel‐Duby, R. , & Olson, E. N. (2010). Myogenin and class II HDACs control neurogenic muscle atrophy by inducing E3 ubiquitin ligases. Cell, 143(1), 35–45. 20887891 10.1016/j.cell.2010.09.004 PMC2982779

[eph13390-bib-0057] Nay, K. , Jollet, M. , Goustard, B. , Baati, N. , Vernus, B. , Pontones, M. , Lefeuvre‐Orfila, L. , Bendavid, C. , Rué, O. , Mariadassou, M. , Bonnieu, A. , Ollendorff, V. , Lepage, P. , Frédéric Derbré, X. , & Koechlin‐Ramonatxo, C. (2019). Gut bacteria are critical for optimal muscle function: A potential link with glucose homeostasis. American Journal of Physiology. Endocrinology and Metabolism, 317(1), E158–E171. 31039010 10.1152/ajpendo.00521.2018

[eph13390-bib-0058] Ni, Y. , Lohinai, Z. , Heshiki, Y. , Dome, B. , Moldvay, J. , Dulka, E. , Galffy, G. , Berta, J. , Weiss, G. J. , Sommer, M. O. A. , & Panagiotou, G. (2021). Distinct composition and metabolic functions of human gut microbiota are associated with cachexia in lung cancer patients. ISME Journal, 15(11), 3207–3220. 34002024 10.1038/s41396-021-00998-8 PMC8528809

[eph13390-bib-0059] Okamoto, T. , Morino, K. , Ugi, S. , Nakagawa, F. , Lemecha, M. , Ida, S. , Ohashi, N. , Sato, D. , Fujita, Y. , & Maegawa, H. (2019). Microbiome potentiates endurance exercise through intestinal acetate production. American Journal of Physiology. Endocrinology and Metabolism, 316(5), E956–E966. 30860879 10.1152/ajpendo.00510.2018

[eph13390-bib-0060] Oray, M. , Abu Samra, K. , Ebrahimiadib, N. , Meese, H. , & Foster, C. S. (2016). Long‐term side effects of glucocorticoids. Expert Opinion on Drug Safety, 15(4), 457–465. 26789102 10.1517/14740338.2016.1140743

[eph13390-bib-0061] Pal, R. , Palmieri, M. , Loehr, J. A. , Li, S. , Abo‐Zahrah, R. , Monroe, T. O. , Thakur, P. B. , Sardiello, M. , & Rodney, G. G. (2014). Src‐dependent impairment of autophagy by oxidative stress in a mouse model of Duchenne muscular dystrophy. Nature Communications, 5(1), 4425. 10.1038/ncomms5425 PMC4101811 25028121

[eph13390-bib-0062] Passamano, L. , Taglia, A. , Palladino, A. , Viggiano, E. , D'Ambrosio, P. , Scutifero, M. , Cecio, M. R. , Torre, V. , de Luca, F. , Picillo, E. , Paciello, O. , Piluso, G. , Nigro, G. , & Politano, L. (2012). Improvement of survival in Duchenne Muscular Dystrophy: Retrospective analysis of 835 patients. Acta Myologica, 31(2), 121–125. 23097603 PMC3476854

[eph13390-bib-0063] Pedersen, B. K. , Steensberg, A. , Fischer, C. , Keller, C. , Keller, P. , Plomgaard, P. , Febbraio, M. , & Saltin, B. (2003). Searching for the exercise factor: Is IL‐6 a candidate? Journal of Muscle Research and Cell Motility, 24(2/3), 113–119. 14609022 10.1023/a:1026070911202

[eph13390-bib-0064] Pedersen, M. E. F. , Fatemian, M. , & Robbins, P. A. (1999). Identification of fast and slow ventilatory responses to carbon dioxide under hypoxic and hyperoxic conditions in humans. Journal of Physiology, 521(1), 273–287. 10562351 10.1111/j.1469-7793.1999.00273.x PMC2269657

[eph13390-bib-0065] Perry, R. J. , Peng, L. , Barry, N. A. , Cline, G. W. , Zhang, D. , Cardone, R. L. , Petersen, K. F. , Kibbey, R. G. , Goodman, A. L. , & Shulman, G. I. (2016). Acetate mediates a microbiome‐brain‐β‐cell axis to promote metabolic syndrome. Nature, 534(7606), 213–217. 27279214 10.1038/nature18309 PMC4922538

[eph13390-bib-0066] Petersen, L. M. , Bautista, E. J. , Nguyen, H. , Hanson, B. M. , Chen, L. , Lek, S. H. , Sodergren, E. , & Weinstock, G. M. (2017). Community characteristics of the gut microbiomes of competitive cyclists. Microbiome, 5(1), 98. 28797298 10.1186/s40168-017-0320-4 PMC5553673

[eph13390-bib-0067] Qiu, Y. , Yu, J. , Li, Y. , Yang, F. , Yu, H. , Xue, M. , Zhang, F. , Jiang, X. , Ji, X. , & Bao, Z. (2021). Depletion of gut microbiota induces skeletal muscle atrophy by FXR‐FGF15/19 signalling. Annals of Medicine, 53(1), 508–522. 33783283 10.1080/07853890.2021.1900593 PMC8018554

[eph13390-bib-0068] Rastelli, M. , Cani, P. D. , & Knauf, C. (2019). The gut microbiome influences host endocrine functions. Endocrine Reviews, 40(5), 1271–1284. 31081896 10.1210/er.2018-00280

[eph13390-bib-0069] Rosenberg, A. S. , Puig, M. , Nagaraju, K. , Hoffman, E. P. , Villalta, S. A. , Rao, V. A. , Wakefield, L. M. , & Woodcock, J. (2015). Immune‐mediated pathology in Duchenne muscular dystrophy. Science Translational Medicine, 7(299), 299rv4. 10.1126/scitranslmed.aaa7322 PMC5951380 26246170

[eph13390-bib-0070] Ryu, D. , Mouchiroud, L. , Andreux lope, A. , Katsyuba, E. , Moullan, N. , Nicolet‐dit‐F, A. A. , Williams, E. G. , Jha, P. , lo Sasso, G. , Huzard, D. , Aebischer, P. , Sandi, C. , Rinsch, C. , & Auwerx, J. (2016). Urolithin A induces mitophagy and prolongs lifespan in *C. elegans* and increases muscle function in rodents. Nature Medicine, 22(8), 879–888. 10.1038/nm.4132 27400265

[eph13390-bib-0071] Sander, M. , Chavoshan, B. , Harris, S. A. , Iannaccone, S. T. , Stull, J. T. , Thomas, G. D. , & Victor, R. G. (2000). Functional muscle ischemia in neuronal nitric oxide synthase‐deficient skeletal muscle of children with Duchenne muscular dystrophy. Proceedings of the National Academy of Sciences, USA, 97(25), 13818–13823. 10.1073/pnas.250379497 PMC17659 11087833

[eph13390-bib-0072] Sandri, M. , Lin, J. , Handschin, C. , Yang, W. , Arany, Z. P. , Lecker, S. H. , Goldberg, A. L. , & Spiegelman, B. M. (2006). PGC‐1 protects skeletal muscle from atrophy by suppressing FoxO3 action and atrophy‐specific gene transcription. Proceedings of the National Academy of Sciences, USA, 103(44), 16260–16265. 10.1073/pnas.0607795103 PMC1637570 17053067

[eph13390-bib-0073] Sasaki, T. , Kuboyama, A. , Mita, M. , Murata, S. , Shimizu, M. , Inoue, J. , Mori, K. , & Sato, R. (2018). The exercise‐inducible bile acid receptor Tgr5 improves skeletal muscle function in mice. Journal of Biological Chemistry, 293(26), 10322–10332. 29773650 10.1074/jbc.RA118.002733 PMC6028981

[eph13390-bib-0074] Scheiman, J. , Luber, J. M. , Chavkin, T. A. , Macdonald, T. , Tung, A. , Pham, L.‐D. , Wibowo, M. C. , Wurth, R. C. , Punthambaker, S. , Tierney, B. T. , Yang, Z. , Hattab, M. W. , Avila‐Pacheco, J. , Clish, C. B. , Lessard, S. , Church, G. M. , & Kostic, A. D. (2019). Meta‐omics analysis of elite athletes identifies a performance‐enhancing microbe that functions via lactate metabolism. Nature Medicine|, 25(7), 1104–1109. 10.1038/s41591-019-0485-4 PMC7368972 31235964

[eph13390-bib-0075] Schepper, J. D. , Collins, F. , Deliz Rios‐Arce, N. , Jun Kang, H. , Schaefer, L. , Gardinier, J. D. , Raghuvanshi, R. , Quinn, R. A. , Britton, R. , Parameswaran, N. , & McCabe, L. R. (2019). Involvement of the gut microbiota and barrier function in glucocorticoid‐induced osteoporosis. Journal of Bone and Mineral Research, 35(4), 801–820. 10.1002/jbmr.3947 31886921

[eph13390-bib-0076] Shin, H. J. R. , Kim, H. , Oh, S. , Lee, J. G. , Kee, M. , Ko, H. J. , Kweon, M. N. , Won, K. J. , & Baek, S. H. (2016). AMPK‐SKP2‐CARM1 signalling cascade in transcriptional regulation of autophagy. Nature, 534(7608), 553–557. 27309807 10.1038/nature18014 PMC5568428

[eph13390-bib-0077] Swann, J. R. , Want, E. J. , Geier, F. M. , Spagou, K. , Wilson, I. D. , Sidaway, J. E. , Nicholson, J. K. , & Holmes, E. (2011). Systemic gut microbial modulation of bile acid metabolism in host tissue compartments. Proceedings of the National Academy of Sciences, USA, 108(1), 4523–4530. 10.1073/pnas.1006734107 PMC3063584 20837534

[eph13390-bib-0078] Ticinesi, A. , Lauretani, F. , Milani, C. , Nouvenne, A. , Tana, C. , del Rio, D. , Maggio, M. , Ventura, M. , & Meschi, T. (2017). Aging gut microbiota at the cross‐road between nutrition, physical frailty, and sarcopenia: Is there a gut‐muscle axis? Nutrients, 9(12), 1303. 29189738 10.3390/nu9121303 PMC5748753

[eph13390-bib-0079] Ubachs, J. , Ziemons, J. , Soons, Z. , Aarnoutse, R. , van Dijk, D. P. J. , Penders, J. , van Helvoort, A. , Smidt, M. L. , Kruitwagen, R. , Baade‐Corpelijn, L. , Olde Damink, S. W. M. , & Rensen, S. S. (2021). Gut microbiota and short‐chain fatty acid alterations in cachectic cancer patients. Journal of Cachexia, Sarcopenia and Muscle, 12(6), 2007–2021. 34609073 10.1002/jcsm.12804 PMC8718054

[eph13390-bib-0080] Valentino, T. R. , Vechetti, I. J. , Mobley, C. B. , Dungan, C. M. , Golden, L. , Goh, J. , Mccarthy, J. J. , & Mccarthy, J. J. (2021). Dysbiosis of the gut microbiome impairs mouse skeletal muscle adaptation to exercise. The Journal of Physiology, 599(21), 4845–4863. 34569067 10.1113/JP281788 PMC8733630

[eph13390-bib-0081] Vantrappen, G. , Janssens, J. , Hellemans, J. , & Ghoos, Y. (1977). The interdigestive motor complex of normal subjects and patients with bacterial overgrowth of the small intestine. Journal of Clinical Investigation, 59(6), 1158–1166. 864008 10.1172/JCI108740 PMC372329

[eph13390-bib-0082] Walsh, M. E. , Bhattacharya, A. , Sataranatarajan, K. , Qaisar, R. , Sloane, L. , Rahman, M. M. , Kinter, M. , & van Remmen, H. (2015). The histone deacetylase inhibitor butyrate improves metabolism and reduces muscle atrophy during aging. Aging Cell, 14(6), 957–970. 26290460 10.1111/acel.12387 PMC4693467

[eph13390-bib-0083] Wang, M. , Zhu, Z. , Lin, X. , Li, H. , Wen, C. , Bao, J. , & He, Z. (2021). Gut microbiota mediated the therapeutic efficacies and the side effects of prednisone in the treatment of MRL/lpr mice. Arthritis Research & Therapy, 23(1), 240. 34521450 10.1186/s13075-021-02620-w PMC8439074

[eph13390-bib-0084] Wang, Y.‐D. , Chen, W.‐D. , Yu, D. , Forman, B. M. , & Huang, W. (2011). The G‐Protein‐Coupled Bile Acid Receptor, Gpbar1 (TGR5), negatively regulates hepatic inflammatory response through antagonizing nuclear factor kappa light‐chain enhancer of activated B cells in mice. Hepatology, 54(4), 1421–1432. 21735468 10.1002/hep.24525 PMC3184183

[eph13390-bib-0085] Watanabe, M. , Houten, S. M. , Mataki, C. , Christoffolete, M. A. , Kim, B. W. , Sato, H. , Messaddeq, N. , Harney, J. W. , Ezaki, O. , Kodama, T. , Schoonjans, K. , Bianco, A. C. , & Auwerx, J. (2006). Bile acids induce energy expenditure by promoting intracellular thyroid hormone activation. Nature, 439(7075), 484–489. 16400329 10.1038/nature04330

[eph13390-bib-0086] Yan, H. , Diao, H. , Xiao, Y. , Li, W. , Yu, B. , He, J. , Yu, J. , Zheng, P. , Mao, X. , Luo, Y. , Zeng, B. , Wei, H. , & Chen, D. (2016). Gut microbiota can transfer fiber characteristics and lipid metabolic profiles of skeletal muscle from pigs to germ‐free mice OPEN. Scientific Reports, 6(1), 31786. 27545196 10.1038/srep31786 PMC4992887

[eph13390-bib-0087] Yan, J. , Herzog, J. W. , Tsang, K. , Brennan, C. A. , Bower, M. A. , Garrett, W. S. , Sartor, B. R. , Aliprantis, A. O. , & Charles, J. F. (2016). Gut microbiota induce IGF‐1 and promote bone formation and growth. Proceedings of the National Academy of Sciences, USA, 113(47), E7554–E7563. 10.1073/pnas.1607235113 PMC5127374 27821775

[eph13390-bib-0088] Zarrinpar, A. , Chaix, A. , Xu, Z. Z. , Chang, M. W. , Marotz, C. A. , Saghatelian, A. , Knight, R. , & Panda, S. (2018). Antibiotic‐induced microbiome depletion alters metabolic homeostasis by affecting gut signaling and colonic metabolism. Nature Communications, 9(1), 2872. 10.1038/s41467-018-05336-9 PMC6054678 30030441

